# Global Analysis of RTS, S/AS01 Malaria Vaccine Acceptance Rates and Influencing Factors: A Systematic Review

**DOI:** 10.7759/cureus.60678

**Published:** 2024-05-20

**Authors:** Farrukh Ansar, Abdullah Azzam, Mohammad S Rauf, Zeeshan Ajmal, Gohar Asad Ullah, Shiza Rauf, Rabia Akram, Fatima K Ghauri, Fizza Chudhary, Hamdah Iftikhar, Ashir Iqbal, Muhammad Bilal Ahmad

**Affiliations:** 1 Department of Medicine, Alkhidmat Raazi Hospital, Rawalpindi, PAK; 2 Department of Medicine and Surgery, Northwest General Hospital, Peshawar, PAK; 3 Department of Anesthesiology and Critical Care, Gulab Devi Hospital, Lahore, PAK; 4 Department of Medicine, Leicester Royal Infirmary, Leicester, GBR; 5 Department of Internal Medicine, Bahawal Victoria Hospital, Bahawalpur, PAK; 6 Department of Internal Medicine, Quaid-E-Azam Medical College, Bahawalpur, PAK; 7 Department of Pharmacology and Therapeutics, Niazi Medical & Dental College, Sargodha, PAK; 8 Department of Medicine and Surgery, Rai Medical College, Sargodha, PAK; 9 Department of Medicine, Quaid-e-Azam International Hospital, Islamabad, PAK

**Keywords:** vaccine hesitancy, global health, public perceptions, vaccine acceptability, malaria vaccine

## Abstract

Malaria remains a significant global health challenge, with Plasmodium parasites transmitted by Anopheles mosquitoes causing substantial morbidity and mortality. Despite historical efforts, malaria continues to affect millions worldwide, particularly in tropical regions. This systematic review aimed to assess the acceptability of the RTS, S/AS01 malaria vaccine among diverse populations. A comprehensive search strategy was employed across databases such as Cochrane Library, Embase, Google Scholar, and Medline. Studies were included based on specific criteria, including observational and cross-sectional designs involving adults. Data extraction and analysis were conducted meticulously, encompassing key variables related to vaccine acceptance rates and influencing factors. Analysis of 18 studies involving 18,561 participants revealed an overall malaria vaccine acceptance rate of 87.51%, ranging from 32.26% to 99.30%. Significant variations were observed based on demographics, with Ghana and Nigeria reporting high acceptance rates. Factors influencing acceptance included knowledge levels, past vaccination experiences, community preferences, and engagement in malaria prevention behaviors. Concerns about adverse reactions and regional disparities were noted as potential barriers to acceptance. This review highlights the importance of understanding public perceptions and concerns regarding malaria vaccines to enhance vaccine coverage and uptake. Tailored communication strategies, advocacy efforts, and targeted education interventions are crucial for addressing misconceptions and increasing vaccine acceptance. Policy recommendations should consider demographic and regional factors to ensure effective implementation of malaria vaccination programs, ultimately contributing to global malaria prevention efforts and public health initiatives.

## Introduction and background

Malaria is caused by infection with Plasmodium protozoan parasites that are spread by female Anopheles mosquitos [1]. Alphonse Laveran discovered malaria parasites in the blood of malaria patients in 1880, which led to our current understanding of the parasites [1]. Since then, humanity has fought this fatal illness, but it remains a serious threat to public health. Malaria is believed to have killed 300 million people in the twentieth century, accounting for 2-5% of all fatalities [1]. Malaria is a significant challenge in tropical areas, including parts of Asia, the Sub-Saharan deserts, and the Amazon basin [2]. However, 40% of the world's population still lives in areas where they are at risk of getting infected by malarial parasites [2]. According to the most recent World Malaria Report, published in December 2021, approximately 600,000 people died in 2020 because of malaria infection [2]. Malaria was also reported to have had the greatest impact in Africa, but its detrimental effects are spreading to Asia and other sub-tropical regions [3].

The development of a vaccine to prevent malaria infection in the general population began in the 1990s. However, in October 2021, the World Health Organization (WHO) authorized a malaria vaccine for the first time [4]. Successful trials on 900,000 children in Ghana, Kenya, and Malawi have led to the final clearance [4]. According to the WHO, the vaccine has a great safety profile, great cost efficiency, and high effectiveness [4]. For comprehensive malaria control, WHO recommended that the RTS, S/AS01 malaria vaccine should be employed for the prevention of Plasmodium (P.) falciparum malaria in children living in high transmission areas [4]. To minimize the burden of malaria infection, RTS, S/AS01, malaria vaccine should be administered in four doses to children from the age of five months [4]. However, vaccination apprehension among the public is a significant impediment to appropriate vaccine administration and coverage. It is critical to assess the public acceptability of the malaria vaccine and to understand their concerns and misunderstandings. If the intervention is intended to address local barriers that exist in diverse people and communities, the acceptance rate can be increased. With the introduction of malaria vaccinations, it is critical to evaluate malaria preventive measures and the community acceptability of malaria vaccines.

This study aims to conduct a systematic literature review to ascertain the prevalence of acceptance of the RTS, S malaria vaccine. The results of this investigation may provide valuable insights for policymakers and other stakeholders, aiding in the identification of suitable public health strategies to enhance vaccine coverage and uptake within malaria-endemic communities.

## Review

Methodology

Research Question

A systematic review was conducted to determine the level of acceptability of the malaria vaccine among the general population.

Search Strategy

**Figure 1:**
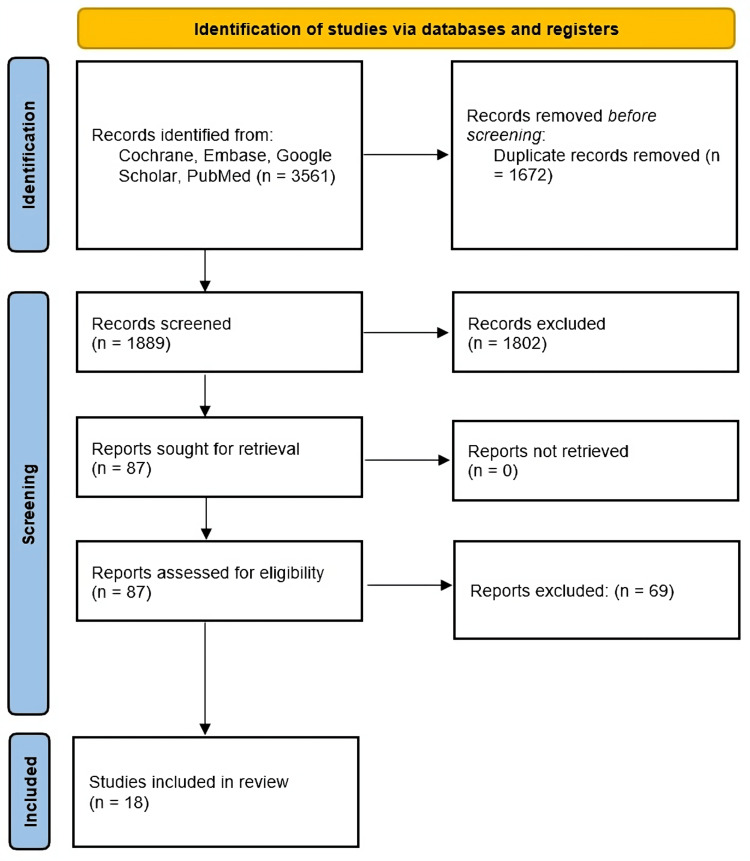
PRISMA flow diagram of literature search PRISMA: Preferred Reporting Items for Systematic Reviews and Meta-Analyses

The databases searched were the Cochrane Library, Embase, Google Scholar, and Medline. These databases were systemically screened through the construction of a search strategy using the medical subject headings (Mesh). A comprehensive search technique was developed and Mesh terms; 'Malaria Vaccine' AND ('Acceptance' OR 'Hesitancy' OR 'Acceptability' OR 'Willingness' OR 'Accept' OR 'Rejection'). Combining the topic heading search with the keyword free-text search yielded a comprehensive search technique. Research papers from inception until March 30, 2024, were included. Figure 1 shows the Preferred Reporting Items for Systematic Reviews and Meta-Analyses (PRISMA) flow diagram of the literature search. The articles found by the search were kept in the Mendeley reference manager. Additional papers were identified by reviewing the reference lists of relevant research. The titles and abstracts of all identified studies were screened, followed by an evaluation of the complete texts of the retained papers to identify eligible research.

Eligibility Criteria

We included all available observational studies and cross-sectional studies, including only quantitative studies in nature. All those studies that involved a human adult population (age of participants over 18 years) were included. Letters to the editors, conference abstracts, case reports and case series, pediatric studies, commentaries, and duplicates were excluded from the finalized pool of studies.

Data Extraction

The full texts of the selected abstracts were acquired and meticulously assessed against our predetermined eligibility criteria. The authors worked independently to extract data from each manuscript. In instances where discrepancies arose between the two authors, the principal author intervened, initiating a comprehensive discussion to reconcile differences and reach a consensus. At this stage, studies lacking crucial data for our research question and subsequent analysis were excluded. A standardized form was employed for data extraction from the shortlisted papers to ensure uniformity. The reviewers independently scrutinized the data-gathering tool, aiming to establish consistency and minimize discrepancies in the process. This meticulous approach to data extraction and review not only ensured the accuracy of the collected information but also laid a solid foundation for the subsequent stages of analysis and synthesis in this systematic literature review. Subsequently, the authors systematically extracted data from eligible studies, utilizing a Microsoft Excel 2016 form (Microsoft Corporation, Redmond, WA, US) as the designated repository. To ensure the utmost precision and reliability, a comprehensive double-checking process was implemented, with another author meticulously reviewing the extracted data. The extracted information encompassed essential variables, including the first author's name, publication year, journal reference, country and location of the study, year of study, study setting and design, sample size, study population, and vaccine acceptance rates. This data extraction procedure was instrumental in fortifying the integrity of the ensuing analysis.

Data Analysis

The extracted data underwent a coding process using IBM SPSS Statistics Version 23 software (IBM Corp., Armonk, NY, US). Various variables were systematically coded, encompassing key parameters such as the number of participants, mean age, and gender distribution. The determination of the overall vaccine acceptance rate was executed by calculating the mean of acceptance rates derived from individual studies. This methodological approach ensures a comprehensive and standardized analysis of the coded variables, facilitating robust statistical assessments and in-depth insights into vaccine acceptance trends across the reviewed studies (Table 1).

**Table 1:** Summary of included studies reporting the acceptance rates of a malaria vaccine

First Author	Year of Study	Sample Size	Willing to vaccinate	Acceptance Rate	Country of Study	Population Type
Ughasoro, Maduka D [5]	2018	156	153	98.07%	Nigeria	Household Heads
Uchechukwu M. Chukwuocha [6]	2018	500	480	96.00%	Nigeria	Mothers
Etokidem AJ [7]	2015	262	139	53.05%	Nigeria	Mothers
Tolulope O Musa-Booth [8]	2020	180	176	97.77%	Nigeria	Mothers
Ilemona AB [9]	2012	427	371	86.88%	Nigeria	Caregivers
Mary Yetunde Ajayi [10]	2023	504	463	91.86%	Nigeria	Caregivers
Abdulmuminu Isah [11]	2023	170	92	54.11%	Nigeria	Pharmacists
Lawrence G Febir [12]	2013	466	361	77.46%	Ghana	Parents/Religious Leaders/Healthcare providers
Dennis Tabiri [13]	2021	424	399	94.10%	Ghana	Caregivers/Parents
Mustapha Immurana [14]	2022	3004	2843	94.64%	Ghana	Mothers/Household Heads
Sally Mtenga [15]	2016	2123	1788	84.22%	Tanzania	Mothers
Idda Romore [16]	2015	5502	5120	93.05%	Tanzania	Mothers
Getachew Asmare [17]	2022	406	131	32.26%	Ethiopia	Caregivers
David I Ojakaa [18]	2014	1997	1699	85.07%	Kenya	Mothers
Kaci D McCoy [19]	2021	615	585	95.12%	Sierra Leone	General Public
Sara E White [20]	2018	143	142	99.30%	Peru	Household Heads
Klara Robl [21]	2023	1277	995	77.91%	Guinea & Sierra Leone	Caregivers
M Ashraful Amin [22]	2023	405	287	70.86%	Bangladesh	Parents
Total	-	18561	16224	87.51%	-	-

Results

Characteristics of Studies Included in This Review

Analysis of 18 studies yielded that the total number of participants was 18561. The majority of the studies were published in the African Region. There were seven studies from Nigeria [5-11], three from Ghana [12-14], two from Tanzania [15,16], and six from other countries [17-22]. The sample population exhibited diversity, encompassing a range of individuals from single mothers to the general public. Specifically, six studies focused exclusively on responses from mothers, two studies were centered on household heads [5,20], and four studies derived data from caregivers [5,10,17,21]. The remaining studies incorporated a combination of various population types.

In terms of the sample size, the highest was 5502 from a Tanzanian study [16], whereas the lowest sample size was reported in a study conducted in Peru, with only 143 participants [20]. The average sample size across studies was 1031. The majority of the studies were published in 2023 (n = 4) while only six studies were published before 2018, with all others published after 2018.

Malaria Vaccine Acceptance Rate

The overall acceptance rate was 87.51% (N=166224), with rates ranging from 32.26% [17] to 99.30% [20]. The study in Peru had the highest acceptance rate [20] while the Ethiopian study reported the lowest [17]. Specifically, Ghana recorded the highest rate of 94.64% [14], whereas Nigeria's highest rate was 98% [5]. Among studies that exclusively sampled mothers, the highest acceptance rate was 97.7% from a Nigerian study [8], with the lowest rate also coming from Nigeria at 53% [7].

Factors Associated With Vaccine Acceptability and Reasons for Hesitancy to Accept the Malaria Vaccine

Asmare and colleagues surveyed caregivers and identified marital status, knowledge levels, and previous experiences with childhood vaccination as significant factors linked with willingness to accept the malaria vaccine [17]. A study conducted among mothers highlighted stakeholders' positive opinions towards the combined use of malaria vaccine and insecticide-treated nets (ITNs), emphasizing sustained high acceptance even with potential limitations of the vaccine [15]. A Nigerian study conducted among household heads found a correlation between malaria prevalence, presumptive treatment practices, low bed net usage, and the necessity of introducing the malaria vaccine. Ojakaa emphasized the need for targeted education, especially in regions with low acceptance rates, among older caregivers, and those with limited literacy [18]. A study conducted on the general population in Sierra Leone observed positive past vaccination experiences and high engagement in malaria prevention behaviors as influencers [19] while another similar study noted community preference for the vaccine over malaria drugs in Central Ghana. Another study underscored near-universal acceptance of a transmission-blocking malaria vaccine in the Peruvian Amazon, emphasizing community-level protection [20]. A Nigerian study reported a high intent to comply with future malaria vaccination among mothers [6], whereas a study from Ghana revealed educational levels, previous adverse reactions, and perceptions about vaccine abundance as factors affecting vaccine uptake among parents [13]. Romore et al. highlighted the complementary role of the malaria vaccine with existing interventions through immunization programs [16] while Etokidem et al. emphasized the importance of advocacy and health education to dispel myths surrounding the vaccine [7]. Researchers from Nigeria highlighted regional variations and the role of knowledge levels in malaria vaccine acceptance and stressed the need for consistent messaging to enhance community knowledge and attitude [8]. An old, unique study from 2012, identified caregiver education levels, spousal influence, and community health worker engagement as crucial factors in vaccine acceptance [9]. On the other hand, a recent study published in 2023 noted inadequate caregiver awareness and recommended tailored communication strategies [10]. Immurana et al. emphasized the significance of targeted awareness efforts considering demographic and socioeconomic factors [14].

Discussion

Individuals' understandings of diseases and the desirable impacts of therapies that aim to prevent diseases, according to the health belief model, are major drivers of people's attitudes toward health interventions [23]. The effective use of community participation is hampered by several factors, including identifying actual stakeholders of interest and evaluating their level of involvement [20].

In our study, we observed an overall acceptance rate of 87.51% for the malaria vaccine, which stands in stark contrast to the pooled prevalence of COVID-19 vaccine acceptance among study participants in Africa and Ethiopia, which were found to be 55.04% and 51.64%, respectively [24,25]. This discrepancy in acceptance rates underscores the complex interplay of factors influencing vaccine acceptance in different contexts. Previous research has pointed to various determinants contributing to vaccine acceptance, including demographic factors such as gender and educational status, as well as knowledge and attitudes toward vaccines [24,25]. Our findings also align with existing literature highlighting the relationship between literacy and vaccine acceptability [13]. A pertinent comparison can be drawn with the acceptance rate of the typhoid conjugate vaccine, introduced in 2019 for typhoid endemic areas, which was reported at 96.2% by Batool et al. [26]. This comparison underscores the influence of disease awareness and perception on vaccine acceptability. It is evident that while factors such as disease prevalence and awareness play a crucial role, the novel nature of COVID-19 has introduced unique challenges to vaccine acceptance. The contrasting histories of malaria, a longstanding burden in Africa, and COVID-19, a recent global pandemic, further emphasize the need for nuanced approaches to understanding and addressing vaccine hesitancy. Additionally, our study echoes the findings of previous research highlighting the enduring impact of childhood exposure to diseases on caregivers' attitudes toward vaccines, suggesting a deeper cultural and experiential dimension to vaccine acceptance [27]. Thus, while the observed variance in vaccine acceptability rates between malaria and COVID-19 is notable, it underscores the multifaceted nature of vaccine acceptance, shaped by a myriad of individual, cultural, and contextual factors [27].

Indeed, religious beliefs have historically played a significant role in shaping vaccine acceptability, as evidenced by the challenges encountered in eradicating polio in regions such as Pakistan and Afghanistan [27]. Despite extensive efforts, including vaccination campaigns, the persistence of polio in these areas underscores the influence of religious factors on vaccine acceptance [27]. The involvement of religious figures, such as pastors, imams, and local religious leaders, in vaccine awareness efforts is crucial in addressing vaccine hesitancy within religious communities [27]. Adopting strategies like the 3-E approach - Engage, Equip, and Empower - for faith leaders as community ambassadors can facilitate effective communication and engagement with religious communities regarding the importance and safety of vaccines [28]. By actively involving religious leaders in vaccine advocacy and education efforts, it becomes possible to address misconceptions, alleviate fears, and promote vaccine acceptance within communities where religious beliefs significantly influence health-related decisions. This highlights the importance of cultural competence and community engagement strategies in designing interventions to enhance vaccine acceptance, particularly in regions where religious beliefs hold considerable sway over public health practices [27].

The route of administration of vaccines indeed plays a significant role in shaping vaccine acceptability, with some individuals expressing apprehension toward injectable vaccines due to fears of paralysis or other adverse reactions [29]. Addressing these concerns through comprehensive community education efforts is essential. Emphasizing the safety profile of vaccines and providing clear information about possible side effects can help alleviate fears and misconceptions [29]. Implementing pre-vaccination campaigns focused on increasing community awareness and promoting the benefits of vaccination, while simultaneously addressing prevalent misconceptions, is crucial in fostering a supportive environment for vaccination uptake [29]. By proactively addressing concerns before they become widespread, such campaigns can effectively mitigate vaccine hesitancy and build trust in vaccination programs. Historically, the failure of an approved vaccine in a region is mostly due to a lack of community participation. In recent years, there has been a growing recognition of the importance of community participation in the implementation of modern clinical research and community-based preventive strategies [30].

A multifaceted approach is necessary to tackle vaccine hesitancy comprehensively and address the diverse needs of communities [30]. This approach goes beyond individual considerations such as vaccine safety, efficacy, cost, and administration, encompassing broader cultural, social, and religious dimensions [30]. It involves engaging with community leaders, religious figures, and cultural influencers to foster consensus and trust in vaccination efforts [30]. By incorporating community perspectives and sensitivities into vaccination strategies, it becomes possible to tailor interventions to the specific contexts and needs of diverse populations [30]. This inclusive approach not only promotes vaccine acceptance but also strengthens community resilience against vaccine misinformation and hesitancy [28]. Ultimately, by recognizing and addressing the multifaceted nature of vaccine hesitancy, public health efforts can effectively promote vaccination as a vital tool in preventing infectious diseases and safeguarding community health [19].

We excluded qualitative studies and studies involving populations under 18 years of age from our analysis. At the time of the study, only the RTS, S/AS01 vaccine was approved; however, in October 2023, the WHO approved the R21/Matrix-M vaccine for rollout in Africa [31]. Our study has not explored awareness among our study population regarding this newly approved vaccine separately. Additionally, it is important to note that the availability of the malaria vaccine is currently restricted to specific regions within Africa, posing challenges for broader acceptance and implementation. Furthermore, the acceptability of malaria vaccines on other continents remains an area requiring further investigation. We acknowledge that our literature search was limited to select databases and may be subject to language bias, as only studies published in English were included.

## Conclusions

This comprehensive review of 18 studies on malaria vaccine acceptance provides valuable insights into the characteristics of the studies, the diverse populations sampled, and the factors influencing vaccine acceptance and hesitancy. The significant sample size, predominantly from the African region, underscores the importance of this research in understanding the attitudes toward malaria vaccination. The high overall acceptance rate indicates a promising outlook for malaria vaccine adoption, with notable variations across different regions and demographic groups. Factors such as knowledge levels, past vaccination experiences, community perceptions, and socioeconomic factors emerge as key influencers of vaccine acceptance. These findings highlight the necessity for targeted education and communication strategies tailored to specific populations to address hesitancy and enhance vaccine uptake. Additionally, the positive reception of malaria vaccination in conjunction with existing interventions emphasizes the potential for integrated approaches to malaria control. Moving forward, sustained efforts in advocacy, health education, and community engagement are crucial for ensuring widespread acceptance and implementation of malaria vaccination programs, ultimately contributing to the global fight against malaria.
